# Prevention for colorectal cancer: from basic research to clinical study

**DOI:** 10.1186/1479-5876-10-S2-A10

**Published:** 2012-10-17

**Authors:** Jingyuan Fang

**Affiliations:** 1 Division of Gastroenterology and Hepatology, Renji Hospital, Shanghai Jiao-Tong University School of Medicine, Shanghai Institution of Digestive Disease, China; 2 Key Laboratory of Gastroenterology & Hepatology, Ministry of Health, China; 3 State Key Laboratory of Oncogene and Related Genes, 145 Middle Shandong Rd, Shanghai 200001, China

## Background

Colorectal cancer (CRC) is third common cancer in world. Both incidence and mortality of CRC in China are increasing. Pooled individual data indicated that the recurrent rate of colorectal adenoma (CRA) underwent the polypectomy is high.

## Methods

Papers in major gastroenterology journals published were retrieved from MEDLINE. The data from my group and other medical centers were also analyzed.

## Results

Folic acid (FA) and butyrate suppress the cell proliferation in CRC cell lines and in mouse. The tumor incidence in DMH group and DMH + butyrate group were 90% and 30%, respectively. And the subgroup of providing FA without precancerous lesions was more effective than that with precancerous lesions.

The data from RCT, Cohort and case-control study showed that many studies report that aspirin decrease the incidence and mortality of CRC in average-risk persons, aspirin and Celecoxib prevent the initial and recurrence of advanced CRA. However, NSAID are ulcerogenic to the stomach and duodenum and lead to a threefold to 10fold increase in ulcer complications, hospitalisation, and death from ulcer disease. Followed along with the increasing the dose of cox2 inhibitors, although prevention effect increased, while the serious cardiovascular events was increased.

Many studies indicated that a dose-response inverse association between dietary folate intake and risk of colon cancer. However, the result from clinical trial published in JAMA 2007 showed that FA at 1mg/d does not reduce CRA recurrent risk, and was associated with higher risks of having 3 or more adenomas and of non CRC. In fact, the FA prevention effect is associated with FA concentration at baseline. For patients who’s FA concentration lower than 7.5ng/mL, FA decrease the recurrence rate of CRA, but not patient who’s FA high than 7.5 ng/ml. A report published in Am J Clin Nutr indicated that folate intake and risk of CRC and CRA: modification by time.

The association between fiber and colorectal neoplasia has been intensively investigated. However, in some studies this topic remains controversial. Many studies showed that calcium and vitamin D reduce risk of CRC. We also found that calcium prevent the recurrence of sporadic CRA.

## Conclusions

The primary prevention for CRC contains the primary and secondary prevention for CRA. Primary prevention also is to reduce the incidence of CRA.

